# A Brush With Danger: A Case Report of a Toothbrush Impalement Injury to the Oral Cavity

**DOI:** 10.1155/crid/8409722

**Published:** 2026-06-29

**Authors:** Rahul Bajaria, Janavikulam Thiruchelvam, Colin Hopper, Abdouldaim Ukwas

**Affiliations:** ^1^ University College London Eastman Dental Institute, London, UK; ^2^ Mid and South Essex NHS Foundation Trust, Basildon, UK

**Keywords:** intraoral foreign body, oral impalement injury, paediatric oral trauma, toothbrush injury

## Abstract

Paediatric oral impalement injuries are uncommon but may be associated with significant morbidity due to the proximity of critical neurovascular structures within the head and neck. We report a case of a 4‐year‐old male who sustained a partial impalement injury to the oral cavity following a fall at home whilst brushing his teeth. The toothbrush head penetrated the right pterygomandibular raphe and became lodged medial to the mandibular ramus. Surgical exploration and removal were performed under general anaesthesia, followed by wound irrigation and primary closure. The patient made an uneventful recovery. This case highlights the importance of cautious initial management, comprehensive assessment and safe surgical removal of intraoral foreign bodies in paediatric patients.

## 1. Introduction

Impalement injuries can be defined as ‘penetration by a large, rigid, blunt‐tipped object that traverses a certain body area in a through‐and‐through fashion and often remains in situ at the time of presentation’ [1]. Partial impalement is a term used to describe non–through‐and‐through injuries, as opposed to complete impalement. Toothbrush‐related injuries represent a recognised mechanism, particularly in young children with limited coordination. Although many injuries appear minor, deeper penetration may result in haemorrhage, infection or neurological complications. The aim of the present article is to describe a case of a toothbrush impalement injury to the oral cavity in a 4‐year‐old child.

## 2. Case Report

**Figure 1:**
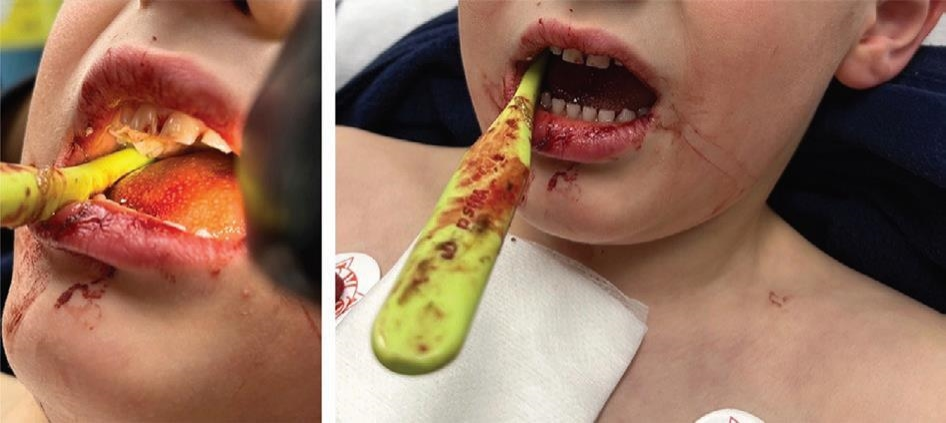
Embedded toothbrush injury to the oral cavity.

A 4‐year‐old male child presented with a toothbrush impalement injury to the oral cavity (Figure 1). The patient′s parent remarked that whilst their son was brushing his teeth at home, he tripped and fell outside the bathroom. The child fell forward, causing the child′s toothbrush to impact the floor, which resulted in its head penetrating the oral mucosa. He remained conscious throughout the event but was visibly distressed as the toothbrush was embedded and lodged in the oral cavity with significant bleeding.

**Figure 2:**
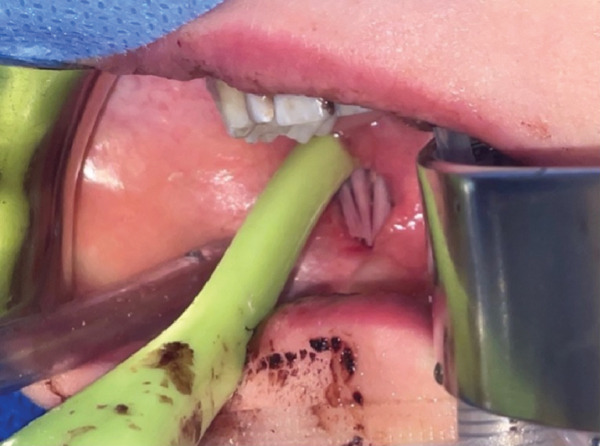
Improved visibility and access under general anaesthesia—toothbrush penetrating the right pterygomandibular raphe.

Upon arrival at the Accident and Emergency Department, the patient was fully conscious with stable clinical observations and no sign of airway or neurological compromise. No further investigations regarding intracranial injury were indicated; however, there was continued mild bleeding from the intraoral wound site. The patient was fit and healthy with no regular medications or drug allergies. Intravenous access was established to administer tranexamic acid and appropriate analgesic medications. Due to the possibility of worsening haemorrhage, the toothbrush was not mobilised, and an emergency referral was made to the Oral and Maxillofacial on‐call team. The entire toothbrush head had pierced the right pterygomandibular raphe and was buried medial to the ramus of the mandible (Figure 2), requiring surgical exploration and removal of the foreign body under general anaesthesia.

**Figure 3:**
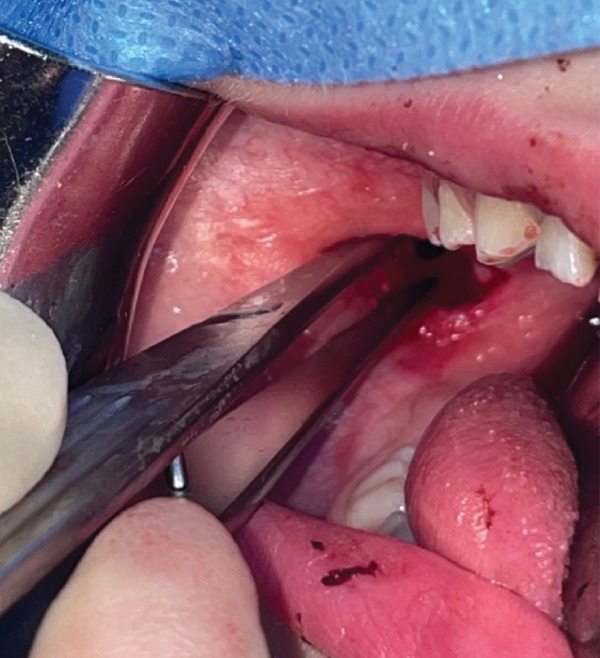
Wound following removal of the foreign body.

Further examination under general anaesthesia allowed greater visibility and access to the oral cavity, which revealed no further soft tissue wounds or dental injuries. The toothbrush was carefully removed (Figure 3), leaving a 3‐cm‐deep gaping wound. The wound was irrigated with saline to ensure no debris remained in situ, and the toothbrush was carefully examined—it was intact with no obvious missing bristles. Oxidised cellulose (Surgicel) was placed inside the wound, and 3‐0 Vicryl Rapide (polyglactin 910) was used to close the wound with simple interrupted sutures. The patient was admitted to the paediatric ward for overnight monitoring and discharged the following day with oral antibiotics (amoxicillin with clavulanic acid) and analgesia (paracetamol) for 1 week. The parents were advised to ensure regular analgesia and a soft diet for 1 week. Recovery was uneventful, and a 4‐week follow‐up showed a complete recovery to normal function.

## 3. Discussion

Penetrating trauma to the head and neck accounts for approximately 1%–2% of all paediatric trauma hospital admissions. Young children aged 2–6 years old are at a higher risk due to a general lack of coordination and a tendency to place objects in the mouth for oral stimulation [2, 3]. Toothbrush impalement injuries are a common (and potentially serious) cause of paediatric oral trauma. Indeed, Kato et al. [4] reviewed 230 paediatric oral soft tissue injuries and found that a toothbrush was the most frequent causative object (32%), followed by a nonspecific stick‐like object (22%) and utensils (12%). Although most injuries are benign, studies have shown that oral impalement injuries can rarely result in life‐threatening complications such as mediastinal emphysema and ischaemic stroke [5, 6].

As with all traumatic injuries, the initial management of impalement injuries should follow the ‘Advanced Trauma Life Support’ protocol, specifically the ‘ABCDE’ approach. Airway assessment and management are the initial objectives during the primary survey. Impaled objects should not be mobilised or removed outside of a controlled environment or prior to airway and neurological assessment and stabilisation. General anaesthesia provides improved access and visibility as well as a safe environment for surgical exploration. There are various anaesthetic considerations at play in paediatric oral impalement injuries, including situations when a protruding object prevents adequate preoxygenation and mask ventilation, thus exposing the patient to a risk of hypoxia. Impaled objects may need to be cut prior to induction, often using rib shears or preferably an ultrasonic scalpel [7]. In the present case, cutting of the object was not required.

Penetrating injury to the oral cavity may involve major vascular structures and deep neck spaces, which can lead to serious complications. In the present case, the anatomical location of injury increased the risk of trauma to branches of the mandibular division (V3) of the trigeminal nerve (CNV), such as the lingual, inferior alveolar and mylohyoid nerves. Although cranial nerve testing is challenging and of limited reliability in young children, we were able to perform basic cranial nerve testing perioperatively, which revealed no sensory or motor deficits. Injury to major blood vessels such as the internal carotid artery or internal jugular vein may cause vessel dissection, transection, thrombosis, pseudoaneurysm or occlusion, which could result in severe haemorrhage and/or neurological deficits [8]. Bacteria introduced into the wound site via a nonsterile toothbrush can proliferate, causing localised inflammation and abscess formation. In severe cases, infection may spread to adjacent tissues, leading to cellulitis, osteomyelitis, mediastinitis or sepsis. It was therefore deemed necessary to provide broad‐spectrum antibiotic prophylaxis in the form of amoxicillin with clavulanic acid, administered intravenously during the inpatient stay followed by oral suspension for 5 days postoperatively.

Imaging may be justified in cases of intraoral foreign body trauma to identify the location of the buried foreign body, including any fragments, and to identify injury to the surrounding tissues, which may not be visible upon clinical examination. In the presented case, we were able to ascertain the size of the wound during intraoral examination, but the depth of the wound could only be estimated by the size of the toothbrush head. It was determined that there was a very low risk of injury to the carotid system or skull base, as the brush head would have had to penetrate significantly deeper for this to be a concern. Bleeding could be encountered from injury to the inferior alveolar vessels, which can often be managed with packing of the area or isolation and ligation. Clinicians should assess the risk of significant vascular or bony injury prior to obtaining imaging. Factors that may inform this assessment include the type of impaled object, the mechanism and velocity of injury and the anatomical site of impalement. The patient in our case was distressed and would likely be uncooperative for imaging techniques, especially those which may require complex positioning, which increases the risk of further accidental injury. Imaging with sedation or general anaesthesia would have likely been required if indicated; however, this was not the case. It was in the patient′s best interests to proceed without imaging or further delay to surgery.

Surgical technique varies significantly depending on the wound site, object involved, depth of penetration and various other factors. Simple removal of objects and wound closure may be possible in less severe impalement injuries. Complex impalement injuries should be guided by the appropriate imaging and may require deep neck exploration, ligation of vessels and/or careful dissection of vital structures to carefully remove impaled objects without causing iatrogenic damage. Haemostatic agents such as oxidised cellulose may be beneficial for nonarterial bleeding. Prior to closure, wounds should be thoroughly inspected for retained foreign bodies and profusely irrigated with antiseptic solution to reduce surgical site infection. This case highlights a successful outcome achieved with prompt assessment, appropriate surgical management and close postoperative monitoring.

Young children should be supervised and positioned in a sedentary, sitting position whilst eating with utensils or brushing their teeth. There is a potential for manufacturers to modify toothbrush design to minimise the risk of injuries, either with a shortened handle, flexible neck or wide protective ring to prevent displacement into the oral cavity upon impact. These modifications should aim at improving the safety of toothbrushing without affecting the ergonomics of the product. The potential for swallowing or choking on the toothbrush and its components should also be considered. In the unfortunate instance of a toothbrush injury, parents should avoid removing or further displacing the foreign body. Urgent medical attention should be sought by calling an ambulance or attending an Accident and Emergency Department.

## 4. Conclusion

Paediatric toothbrush impalement injuries, although uncommon, require careful assessment and management due to the potential for life‐threatening complications. Embedded foreign bodies should not be removed outside a controlled surgical environment, and early specialist involvement is essential. This case demonstrates that prompt adherence to trauma principles, appropriate surgical exploration under general anaesthesia and vigilant postoperative monitoring can result in excellent clinical outcomes. Increased parental supervision during toothbrushing and consideration of safer toothbrush design may help reduce the incidence of such injuries.

## Author Contributions

**Rahul Bajaria:** conceptualisation, writing – original draft. **Abdouldaim Ukwas:** writing – review and editing, supervision. **Colin Hopper:** supervision. **Janavikulam Thiruchelvam:** supervision.

## Funding

No funding was received for this manuscript.

## Disclosure

All authors have read and approved the final version of the manuscript.

## Ethics Statement

Ethical approval was not required for this single anonymised case report, and informed consent was obtained.

## Consent

The child′s parent provided consent for publication of the clinical information and photographs.

## Conflicts of Interest

The authors declare no conflicts of interest.

## Data Availability

All data are available as part of the published article, and no additional source data are required.
